# Cervical spine injuries requiring surgery in a Level I trauma centre in a major German city

**DOI:** 10.1007/s00701-021-05029-1

**Published:** 2021-10-26

**Authors:** Roslind Karolina Hackenberg, Paul Stoll, Kristian Welle, Jasmin Scorzin, Martin Gathen, Charlotte Rommelspacher, Koroush Kabir

**Affiliations:** 1 Department of Orthopedic and Trauma Surgery, University Hospital Bonn, Venusberg-Campus 1, 53127 Bonn, Germany; 2 Department of Hand, Plastic and Reconstructive Surgery, Burn Center, BG Trauma Center Ludwigshafen, University Hospital Heidelberg, Ludwigshafen, Germany; 3 Department of Neurosurgery, University Hospital Bonn, Bonn, Germany

**Keywords:** Cervical spine, Incidence, Injury, Surgery, Neurology

## Abstract

**Background:**

Cervical spine injuries (CSI) are rare in trauma patients, at about 9.2–16.5/100,000 inhabitants in Scandinavia and Canada, and the annual incidence of CSI surgeries in Norway is around 3.0/100,000 inhabitants. However, despite their rarity, the incidence of CSI has increased, thereby assuming an increasing need for surgery. Outside of Scandinavia, no data about the incidence of CSI and subsequent surgeries exist. Therefore, this study aimed to analyse CSI epidemiology and surgery in a German city with a Level I trauma centre both to understand the injury and improve needs–based planning.

**Methods:**

This retrospective, monocentre study included all patients who presented with CSI from 2012–2017 at a university hospital with a Level I trauma centre in a major German city and had permanent residency within the city. Based on the assumption that the patients represented all CSI injuries in the city, as they were treated at the only available Level I trauma centre, the annual incidence of surgeries and neurologic deficits due to CSI were calculated.

**Results:**

A total of 465 patients with 609 CSI were identified. Of these patients, 61 both received surgery and resided in the city (mean age, 68.1 ± 18.3 years; 26 female, 35 male). The incidence of CSI surgeries was calculated as 3.24/100,000 person years (1.75/100,000 in the upper and 1.54/100,000 in the subaxial cervical spine). Neurologic deficits occurred in 0.64/100,000 person years. The incidence of both surgeries and neurologic deficits showed no significant changes over the 6-year study period.

**Conclusions:**

Compared to Scandinavia, an increasing annual incidence for CSI surgeries and neurologic deficits were found. For long-term demand planning with adaptability to demographic changes, cross-regional studies including long-term follow-up are necessary.

## Introduction

Cervical spine injuries (CSI) are a rare entity among trauma and polytrauma patients [4]. In Scandinavia, the annual incidence of cervical spine fractures was found to be 9.2–15/100,000 inhabitants [2, 6]. Additionally, soft tissue injuries of the cervical spine were found in 1.5–8/100,000 inhabitants each year [2, 6]. Furthermore, the overall incidence of CSI was estimated to be 16.5–17.2/100,000 inhabitants per year in Norway and Sweden [2, 6] and 12/100,000 inhabitants per year in Canada [6, 8]. The annual incidence of CSI hospital admissions in China was found to be 65/100,000 patients [16]. However, despite their rarity in relation to the general population and all patients, the incidence of traumatic CSI has significantly increased over the last few decades [9, 13]. In 2005, 4.1% of all trauma patients in the USA suffered from CSI; this rate significantly increased to 5.4% in 2013 (*p* < 0.001) [13]. Another study showed that 6.7% of all polytraumatized patients in the USA present with CSI [4].

The age distribution and gender ratio of CSI have also changed. In 1992, the mean age of patients with CSI was 20–29 years in the UK [15]. In the USA, the mean age had increased to 59 years in 2013 [13], and the incidence of CSI in Norway has significantly increased with increasing age [6, 7]. Furthermore, although the gender ratio of CSI favours men, at 1.5–4.3:1 [2, 4, 10, 13, 16], the amount of women presenting with CSI has significantly increased, specifically with increasing age [2, 9].

Of all CSI, 18–33.5% require surgery [6, 7, 13]. In Norway, the annual incidence of CSI surgeries was estimated to be 3.0–3.1/100,000 inhabitants [6, 7]. However, due to the increasing incidence and changing demographics of CSI, the need for CSI surgeries will likely increase as well. Outside of Scandinavia, though, no data exist concerning CSI incidence, specifically their need for surgery and the rate of coexisting neurologic deficits. This knowledge is not only important for understanding the injury but also essential for needs-based planning. Therefore, this study aimed to analyse CSI epidemiology and need for surgeries in a major German city with one Level I trauma centre.

## Methods and materials

This retrospective, monocentre study included all patients who presented with and received surgical treatment for traumatic CSI at the Level I trauma centre between January 2012 and December 2017. The centre is in the only maximum medical care hospital in the city that has departments of orthopaedic and trauma surgery and neurosurgery. The inclusion criteria were as follows: one or more injuries in the upper and/or subaxial cervical spine, which were assured via X-ray, computed tomography, or magnetic resonance imaging; a recent trauma; and permanent residency in the city of Bonn. The upper cervical spine was defined as the levels C0–C2 and the subaxial cervical spine was defined as the levels C3 to C7. Patients with residence outside of the city and without an osseous or ligamentous CSI, adequate trauma history, or surgical treatment were excluded. Adequate trauma was defined as follows: (1) a traffic accident with a change of velocity of at least 30 km/h that included motor vehicles, motorcycles, bicycles, scooters, and/or pedestrians; (2) a fall, including falls from a height of at least 1 m and staircase falls; and (3) low-energy trauma with a direct impact to the head, including falls from a height of less than 1 m and sports accidents. Patients with an isolated spinal cord injury but without an osseous or ligamentous injury, pathologic fractures, or osteoporotic insufficiency fractures were also excluded.

The regional trauma network included one university hospital, which was a maximum medical care hospital with a Level I trauma centre. The network also included four hospitals with a Level III trauma centre and four hospitals with a Level IV trauma centre. As per regulations within and between the regional trauma networks, patients were transferred to the closest available and most appropriate trauma centre. Besides the hospital included in the present study, the nearest maximum medical care hospitals with Level I trauma centres were at a distance of 30 km, 32 km, 54 km, and 80 km. Due to potential overlap of the rural catchment area, only patients with residency in the city of Bonn were selected and included.

Age, gender, localization of the injury, neurologic deficits, method of admission (i.e., immediate presentation through self-initiative or emergency medical service or transfer from a peripheral hospital), length of hospital stay in our institution, and treatment were documented from the patient records. Based on the assumption that all patients with severe CSI, such as those with neurologic deficits or an indication for surgery, presented and were treated at the only available centre in the city, the annual incidence of traumatic CSI with neurologic deficits and those necessitating surgery was calculated using the number of inhabitants of the city of Bonn. Descriptive statistical analysis was performed using SPSS Statistics, Version 25.0 (IBM, Armonk, NY, USA). The level of significance was defined at *p* < 0.05.

The study was approved by the local ethics committee of the University Hospital Bonn (local review board number 250/18) and performed in accordance with the ethical standards of the institutional and national research committee and the 1964 Helsinki declaration and its later amendments.

## Results

### Study population

Between January 2012 and December 2017, a total of 465 patients with 609 CSI were treated at the university hospital, 130 of whom had permanent residence within the city. Of the 130 patients with a mean age of 67.5 ± 20.4 years, 61 received surgery (46.9%), while 58 received conservative treatment (44.6%) with a cervical collar for an average of 6 weeks, and 11 did not need a specific treatment (8.5%), because their injury had no risk for instability or non-union. The incidence of CSI surgeries was calculated by means of the 61 patients who received surgical treatment.

**Fig. 1:**
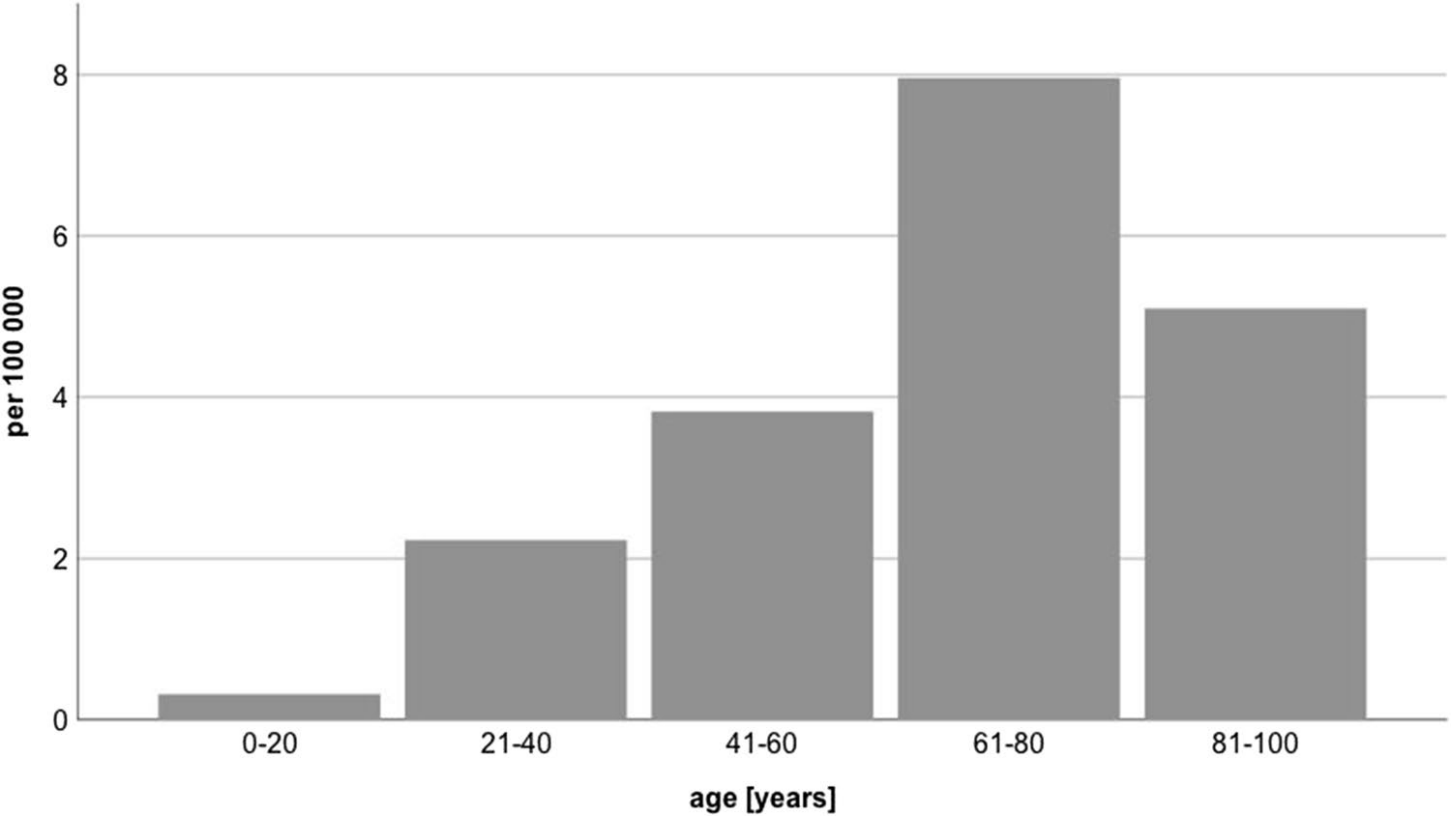
Incidence of surgeries in cervical spine injuries according to age per 100.000 person years

Table 1 displays the patients’ characteristics. The mean age was 68.1 ± 18.3 years (range, 20–90 years). Of the patients, 26 were female (42.6%), and 35 were male (57.4%). The gender ratio was 1.35:1 (male: female). Both patient age and gender distribution showed no significant changes during the 6-year study period (*p* > 0.05). However, the elderly, especially patients with an age of > 60 years, suffered more often from CSI and had a higher risk for CSI necessitating surgery with patients between 61 and 80 years representing the group at highest risk (Fig. 1). Of the patients, 34 (55.7%) had an immediate presentation, either by self-initiative or via emergency medical services; 27 (44.3%) were transferred from a regional hospital for treatment. The mean length of hospital stay was 23.0 ± 20.3 days and showed no significant changes during the six-year study period (*p* > 0.05).

**Table 1:** Patient characteristics in CSI surgery

Year		Total	Ø/year
Number of patients [*n*]		61	10.17
Age [*y*; SD]		68.07 ± 18.33 (min. 20; max. 92)	
Gender [*n*]	Male	35	5.83
	Female	26	4.33
Method of admission [*n*]	Immediate presentation	34	5.67
	Transfer	27	4.50
Length of hospital stay [*d*; SD]		22.97 ± 20.25 (min. 5; max. 88)	
Localisation [*n*]	Upper cervical spine	32	5.33
	Subaxial cervical spine	28	4.67
	Combined	1	0.17
Neurology [*n*]	None	50	8.33
	Radiculopathy	4	0.67
	Incomplete spinal cord injury	3	0.50
	Complete spinal cord injury	4	0.67

### Incidence

On average, 10.2 patients in the city of Bonn suffered from CSI that were treated surgically each year. With an average number of 314,000 citizens, this resulted in an incidence of 3.24/100,000 person years for traumatic CSI receiving surgery. No significant changes in the incidence of CSI surgeries were observed during the study period (*p* > 0.05).

### Neurology

Of the 61 patients, 11 had neurologic deficits because of their CSI; four suffered from a radiculopathy with emphasis on the upper extremities, three suffered from incomplete spinal cord injury, and four suffered from complete spinal cord injury. On average, two patients suffered from neurologic deficits accompanying their CSI each year. Therefore, an incidence of 0.64/100,000 person years for neurologic deficits due to CSI was determined (Table 2). Furthermore, complete spinal cord injury due to CSI was found in 0.21/100,000 person years. No significant increase or decrease was determined in the incidence of neurologic disabilities accompanying CSI throughout the study period.

**Table 2:** Incidence of CSI surgeries and concomitant neurologic deficits in the city of Bonn over a 6-year study period

	Total/year [*n*]	Incidence [100,000 person years]
Inhabitants	314,000 (min. 309,000; max. 320,000)	
CSI surgeries	10.17	3.24
CSI surgeries in the upper cervical spine (C0-C2)	5.50	1.75
CSI surgeries in the subaxial cervical spine (C3-C7)	4.83	1.54
CSI with neurologic deficits	2	0.64

Of the 130 patients with CSI and a residency in Bonn being treated at the Level I trauma centre, 12 patients suffered from spinal cord injury with neurologic deficits. Based on the assumption that all patients with CSI and neurologic deficits in Bonn presented at our institution, the rate for neurologic disabilities in CSI is calculated to be < 9.2%.

### Localization of injury

Of the surgically treated CSI, 32 were located in the upper cervical spine, while 28 were located in the subaxial cervical spine. One patient suffered from a combined injury in the upper and subaxial cervical spine that needed surgery. Therefore, the average annual rate was 5.5 injuries in the upper cervical spine and 4.8 injuries in the subaxial cervical spine that were treated with surgery, including combined injuries. Combined injuries receiving surgery averaged 0.17 patients/year. For surgically treated injuries in the upper cervical spine, an incidence of 1.8/100,000 person years was found, while the incidence for surgically treated injuries in the subaxial cervical spine was 1.5/100,000 person years (Table 2). The incidence of combined injuries in the upper and subaxial cervical spine was 0.054/100,000 person years. On average, 14% more surgeries were performed on account of injuries in the upper cervical spine than the subaxial cervical spine. There was no significant difference in the incidence concerning the localization of injury during the observation period.

## Discussion

The present study was the first to assess the incidence of CSI surgeries as well as the incidence of neurologic deficits due to CSI, for a major German city. The annual incidence of CSI is 17.2/100,000 inhabitants in Sweden [2], 12/100,000 in Canada [6, 8], and 11.8–16.5/100,000 for Norway [6, 7]. Unlike the present study, studies of Scandinavian and Canadian patients also included those with a spinal cord injury without additional osseous or ligamentous injuries of the cervical spine, whereas these patients were excluded from the present study.

Both the mean age of all patients who presented with CSI in the city of Bonn (67.5 years) as well as that of those who received surgery (68.1 years) were higher than the findings of previous studies, in which the mean age and median were between 54 and 56 years [6, 7, 13]. The higher mean age in the present study may have been due to the more balanced gender ratio, which was 1.03:1 (male: female) for all 130 patients and 1.35:1 (male: female) for those who received surgery. In contrast, studies by Fredo et al. (2012 and 2014) found the percentage of men with CSI to be 68% and 69%, respectively [6, 7], which resulted in a male:female ratio of 2.13–2.23:1 in Norway. However, the gender distribution in the USA in 2018 was 1.5:1 (male:female) [13], which was closer to the ratio found by the present study. Brolin et al. (2002) showed that, despite an average ratio of 2.3:1 (male:female), this ratio had dropped from 3.1:1 in 1987 to 1.7:1 in 1999; therefore, the number of women with CSI increased considerably [2]. Not only did the incidence of CSI increase with increasing age, specifically for women, whose average was higher than that of men [6, 7], but the incidence of CSI in women older than 65 years in Sweden was also more than twice as high as the incidence in the general population [2]. Therefore, the greater number of women in the present study may have accounted for the comparatively high mean age.

In the USA, 33.5% of all CSI are treated with surgery, 17.2% of which receive an open stabilisation [13]. In Norway, the number of CSI treated with surgeries was stated as 26.6% [7] and 18% [6]. Conservative treatment was performed in 68.7% of the CSI, while 4.7% required no treatment [7]. The annual incidence of CSI surgeries was estimated to be 3.0/100,000 inhabitants [6], which was concordant but slightly lower than the rate of occurrence in Bonn found by the present study (3.24/100,000 person years). In contrast to studies of Scandinavia and North America, the present study could not determine the number of simple injuries necessitating no or conservative treatment that were, therefore, treated on an outpatient basis or in a peripheral hospital without our knowledge. Thus, the incidence for CSI could not be calculated by the present study. It also has to be mentioned that the amount of conservatively treated or not treated CSI only represents the rates of the Level 1 trauma centre itself. On account of these numbers, it is only valid to conclude that the rate of patients being treated non-surgically for CSI is ≥ 53.1%, while the percentage of CSI needing surgery is ≤ 46.9% being conform with the international average for CSI needing surgery (18–33.5%) [6, 7, 13].

The slightly higher demand for CSI surgeries in the present study as compared to the study in Norway may have been due to a lower threshold for surgery as a treatment for CSI in Germany. An international comparison showed that German spine surgeons chose surgery in 94.2% of CSI patients and significantly more often (*p* < 0.05) than their colleagues in the Netherlands who decided upon surgery in only 58.1% [14]. The tendency to perform surgery more often in the present study may have been due to a lower threshold, but it could also have been due to the higher number of elderly and female patients with CSI. Because of a lower bone quality in elderly women and higher risk of insufficient fracture healing, surgery can prevent secondary complications and non-unions. For further clarification of this aspect, additional research is necessary.

Neurologic deficits occurred with an incidence of 0.64/100,000 person years in the present study, and the incidence of complete paraplegia was 0.21/100,000. Since not all CSI in Bonn were captured in this study, the total rate of neurologic deficits in CSI and the rate of CSI without neurologic deficits could not be determined. Based on the included patients and the total number of patients (130) identified in the present study, with 12 of the 130 patients suffering from neurologic deficits, the rate could be assumed to be < 9.2%. In 1996, Hu et al. found a rate of 77% for CSI without neurologic deficits [8], while Fredo et al. found a rate of 15% with and 79% without neurologic deficits in 2012 [7] resulting in an annual incidence of CSI with neurologic deficits of 1.2/100,000 inhabitants. In a further study, Fredo et al. (2014) found neurologic deficits in CSI in only 4.7% of patients and isolated cervical spinal cord injuries without vertebral injuries in 12.7% of patients [6]. Furthermore, Passias et al. (2018) found an association between neurologic deficits and injuries in the subaxial cervical spine (upper cervical spine, 5%; subaxial cervical spine, 6.7%, respectively) [13]. The consideration with a division into the upper and subaxial cervical spine, as well as spinal cord injuries with and without osseo-ligamentous lesions, may explain the low incidence of accompanying neurologic deficits in the present study. With this knowledge, the rates of accompanying neurological deficits stated in previous studies (34–65%) [2, 11, 16] seem high. Similarly, the amount of complete spinal cord injuries in patients with CSI in the present study (< 3.1%) was almost as low as that found by Fredo et al. in 2012 (2%) [7] and considerably lower than that found by Leucht et al. in 2009 (19.7%) [11]. The low incidence of neurologic deficits and complete spinal cord injuries in the present study may have been a result of the greater number of women and of injuries in the upper cervical spine. Injuries of the upper cervical spine occur specifically in elderly women and are usually caused by low-energy trauma [12]. These factors, as well as the increased width of the spinal canal in the upper cervical spine, which better compensates stenoses, reduce the risk for severe neurological damage [5].

Studies in the USA found a mean length of hospital stay of 8.63 days [13] and 9.6 ± 12 days [4]. In contrast, a Swedish register study found that > 92% of patients with CSI and > 96% of patients with cervical soft tissue injuries had a mean length of hospital stay of ≤ 30 days [2]. Thus, > 35% of patients with CSI and > 25% of patients with soft tissue injuries in the neck had a mean length of hospital stay of 8–30 days, which was similar to that of patients receiving surgery in the present study (23.0 ± 20.3 days). Healthcare systems in central Europe, the USA, and even within Europe differ, possibly resulting in different hospitalisation durations. For instance, the reduced use of halo fixators and preferred use of open surgery in Europe could result in longer hospitalizations than in the USA [6].

This study was the first to calculate the incidence of surgeries as well as neurologic deficits due to CSI in a major German city. The limitations of the study were that only patients with a permanent residence within the city and who were treated in the only available local university hospital were included. Hence, the stated incidence can only be considered an estimation. Patients that only visited the city or worked in the city but had residency outside of the city were not included. Because of overlapping rural catchment areas with neighbouring Level I trauma centres and maximum medical care hospitals, the overall incidence of CSI surgeries in Bonn may have been underestimated. Additionally, injuries that were treated on an outpatient basis or in peripheral hospitals and classified as stable, thereby necessitating no or conservative therapy, could not be detected.

Furthermore, patients who died in the preclinical phase due to trauma and therefore never received hospital treatment could not be detected. Post-mortem studies have shown that 21–24% of patients who die at the site of the accident have CSI with spinal cord injuries [1, 3]. In a register study, Fredo et al. (2014) estimated that 10% of CSI were undetected and therefore assumed an actual annual incidence for CSI of 18/100,000 inhabitants in Norway [6]. Due to the different healthcare systems, the amount of undetected CSI in the present study may have been even higher, potentially 30%. The incidence of surgeries for CSI, however, was not affected by undetected CSI. Still, to display the incidence of CSI and their need for surgical treatment, further studies including patients treated in peripheral hospitals and on an outpatient basis are needed.

## Conclusion

Based on calculations in a German city, the annual need for surgeries to treat CSI and the concurrent neurologic deficits were assessed, providing insights into the local supply situation for care research. While both rates showed no significant changes over a six-year study period, the incidence of CSI surgeries as well as the mean age and rate of women was higher in Germany compared to other studies. For long-term demand planning with adaptability to demographic changes, further cross-regional or register studies including long-term follow-up are necessary.

## Data Availability

Not applicable.

## Abbreviation

CSI: Cervical spine injuries
